# Demixing microwave signals using system-on-chip photonic processor

**DOI:** 10.1038/s41377-024-01404-6

**Published:** 2024-02-27

**Authors:** Sheng Gao, Chu Wu, Xing Lin

**Affiliations:** 1 Department of Electronic Engineering, Tsinghua University, 100084 Beijing, China; 2 Beijing National Research Center for Information Science and Technology, Tsinghua University, 100084 Beijing, China

**Keywords:** Integrated optics, Silicon photonics, Microwave photonics

## Abstract

The integrated photonic processor, co-packaged with electronic peripherals, is proposed for blind source separation of microwave signals, which separates signal-of-interest from dynamic interference with real-time adaptability.

Photonic integrated circuits (PICs) use photons instead of electrons as information carriers that can be utilized for processing wireless signals with tens of GHz bandwidth, light-speed transmission, and low energy loss^1^. PICs use the principle of light-matter interaction to achieve the modulation of different dimensional information of photons, such as amplitude and phase. Previous research has developed cascaded Mach–Zehnder interferometer (MZI) arrays^2,3^ or layers of metastructures^4^ as the modulation elements, micro-ring resonators (MRR) for wavelength-division-multiplexing (WDM) modulation^5,6^, and phase-change materials for non-volatile optical modulation^7^. Existing PICs can only work in a laboratory environment, relying on bulky devices such as oscilloscopes for signal analysis and optical parameter adjustments. Co-packaging with electronic peripherals can enable PICs to achieve online learning and real-time performance for real-world deployments and dynamic scenarios.

Real-time source signals and beam separation are essential for various applications, including 5G wireless communications, radar interference suppression, free-space optical communications, etc. In an electromagnetic environment with limited spectrum resources, extracting the signal-of-interest (SOI) from dynamic radio frequency (RF) interferences in real-time is crucial for ensuring the functionality of wireless devices. Blind source separation (BSS) can separate multiple unknown source signals by processing received multi-channel data without prior information. However, the inherent narrow bandwidth of traditional electronic BSS and the high latency and energy consumption of digital signal processing make it challenging to separate source signals in real-time. Programmable microwave photonic processors can replace traditional RF circuits to process RF signals in the optical domain, greatly improving system bandwidth and response speed while reducing energy transmission losses. Although previous research has implemented basic filtering, time domain integration and difference functions, and high-dimensional signal processing^8,9^, the current system still confronts significant challenges of online adaptability due to the lack of electronic peripherals for analyzing data and adjusting optical parameters of PIC in real time.

A newly published research work in *Light: Science & Applications*, led by Prof. Paul R. Prucnal from Princeton University, proposes a system-on-a-chip microwave photonic processor for real-time BSS with a picosecond delay, which co-packages the fully integrated MRR-based PIC with electronic peripherals to effectively suppress dynamics RF interference^10^. The proposed photonic BSS integrates electro-optical (EO) modulators, MRR weight banks, and balanced photodetectors (BPD). The electronic peripherals are based on a field-programmable gate array (FPGA) chip for high-throughput kurtosis calculation, Nelder–Mead (NM) BSS method, and PIC control, allowing rapid demixing with a refresh rate of 305 Hz by minimizing the inter-device communication. With the calculated demixing matrix, the proposed photonic BSS further incorporates the dithering method to control the weight of the MRR precisely, achieving a processing delay of 15 picoseconds according to the light propagation time. This palm-sized photonic BSS can significantly reduce communication bit error rates and extract SOI with a high signal-to-noise ratio (SNR) in dynamic interference scenarios of mobile communications and radar altimeters. The proposed photonic BSS is capable of real-time adaptability and online learning, which allows it to address dynamic interference problems in complex electromagnetic interference environments.

Figure 1a shows the schematic of the proposed photonic BSS and co-packaged electronic peripherals. The researchers successfully demonstrated that the photonic BSS with two signal processing links could achieve real-time adaptive demixing of the source signal and dynamic interference under the 5G cellular frequency band. Two EO modulators modulate the received RF mixed inputs onto two lasers with different wavelengths, respectively, that coincide with the resonant frequencies of the modulators. After the modulated light has been evenly divided, two MRR weight banks implement the demixing matrix weighting with WDM. The developed MRR has an excellent performance of 19.2 GHz bandwidth and 9-bit weighting accuracy. The weighted optical signals are converted back to the electrical domain by the BPD and then passed through a transimpedance amplifier to produce two amplified outputs. One of these outputs is fed to the electronic peripherals to perform real-time online learning for weight adjustment. The FPGA can sample the PIC output signals in real-time with analog-to-digital conversion and calculate the kurtosis as the objective function. Then the FPGA executes the NM method to iteratively update the demixing weights and applies thermal tuning current by controlling the MRR driver. The adopted dithering method precisely measures the effective weights of the MRR, eliminating the error between the optimized weights and the applied current. The NM method converges rapidly in a mere ten iterations (33 ms) to obtain the demixed signals. From Fig. 1b, it was demonstrated that the photonic BSS with a refresh rate of 305 Hz can successfully recover the source signal from the two-channel received mixtures with a low bit error rate, even when the source signal and dynamic interference are binary phase shift keying modulated signals share the same frequency and data rate.

**Fig. 1:**
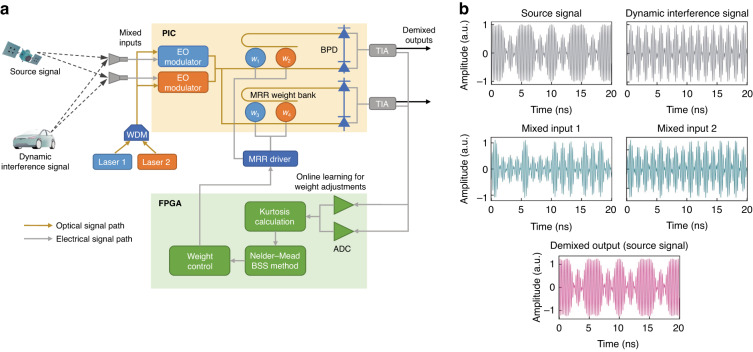
PIC co-packaged with FPGA for real-time BSS of microwave signals. **a** Schematic of the photonic BSS and co-packaged electronic peripherals. **b** Separation demonstration of the source signal and dynamic interference, both of which are BPSK-modulated signals with the same frequency and data rate. a.u. arbitrary unit

This work presents an innovative exploration towards achieving real-time adaptive and intelligent PIC, developing online learning, and adjustment of photonic weights with a millisecond updating rate. Looking forward, PICs expect to achieve real-time modulation and monitoring of more dimensional information of photons, including amplitude, phase, optical mode, and polarization. Furthermore, designing a large-scale MRR weight network can increase the number of separable source signals and make full use of the inherent WDM parallelism of MRR. However, these endeavors may increase the design complexity and systematic errors. To address these challenges, adaptive training algorithms can be employed to train large-scale photonic neural networks and eliminate the mismatch between simulation and experiment^11,12^.

The powerful signal processing capabilities of PIC are not limited to the microwave frequency band, which can also be extended to the optical communication spectrum. Researchers from Politecnico di Milano have proposed an adaptive multi-beam receiver using the MZI-based programmable PIC^13^. This receiver can effectively separate two overlapped arbitrary free-space optical beams with negligible crosstalk in the optical domain. These unknown-shaped beams can share wavelength and polarization, with only orthogonality in directions or spatial modes. This receiver integrates a two-dimensional optical antenna array to couple free-space beams. Then, it modulates the phase and amplitude of the optical signal through the MZI network to achieve the separation of multiple beams. This system can also be designed to automatically configure optimal communication channels without the need for external calculations and calibrations^14^. This method enables a seamless connection between free-space optics and on-chip signal processing. It demonstrates robustness against parametric perturbations and obstacle scattering, which has great advances for multi-beam spatial division multiplexing in free-space optical communication.

It is foreseeable that the development of PICs with online adaptability will bring exciting inspiration for research areas of both microwave photonics and wireless communication. Programmable PICs are expected to realize more complex wireless signal processing algorithms, such as adaptive filtering, spatial spectrum estimation, feature extraction, Fourier transform, coding, and decoding, with unparalleled advantages in computing performance and innovative electronic processors with a new computing paradigm.

## References

[1] Bogaerts W (2020). Programmable photonic circuits. Nature.

[2] Shen YC (2017). Deep learning with coherent nanophotonic circuits. Nat. Photonics.

[3] Pai S (2023). Experimentally realized in situ backpropagation for deep learning in photonic neural networks. Science.

[4] Yan T (2022). All-optical graph representation learning using integrated diffractive photonic computing units. Sci. Adv..

[5] Tait AN (2014). Broadcast and weight: an integrated network for scalable photonic spike processing. J. Lightwave Technol..

[6] Zhang WP (2023). Broadband physical layer cognitive radio with an integrated photonic processor for blind source separation. Nat. Commun..

[7] Feldmann J (2021). Parallel convolutional processing using an integrated photonic tensor core. Nature.

[8] Marpaung D, Yao JP, Capmany J (2019). Integrated microwave photonics. Nat. Photonics.

[9] Dong BW (2023). Higher-dimensional processing using a photonic tensor core with continuous-time data. Nat. Photonics.

[10] Zhang WP (2024). A system-on-chip microwave photonic processor solves dynamic RF interference in real time with picosecond latency. Light Sci. Appl..

[11] Zheng ZY (2023). Dual adaptive training of photonic neural networks. Nat. Mach. Intell..

[12] Lin X (2022). Artificial intelligence built on wireless signals. Nat. Electron..

[13] Milanizadeh M (2022). Separating arbitrary free-space beams with an integrated photonic processor. Light Sci. Appl..

[14] SeyedinNavadeh S (2023). Determining the optimal communication channels of arbitrary optical systems using integrated photonic processors. Nat. Photonics.

